# Entrapment of a Cytotoxic Drug into the Crystal Structure of Calcite for Targeted Drug Delivery

**DOI:** 10.3390/ma14226735

**Published:** 2021-11-09

**Authors:** Amina Vazda, Michael Pujari-Palmer, Wei Xia, Håkan Engqvist

**Affiliations:** Department of Materials Science and Engineering, Division of Applied Materials Science, Uppsala University, 75121 Uppsala, Sweden; amina.vazda@angstrom.uu.se (A.V.); michael.palmer@angstrom.uu.se (M.P.-P.); wei.xia@angstrom.uu.se (W.X.)

**Keywords:** calcite, target drug delivery, pH-responsiveness, in vitro, MCF-7

## Abstract

Controlled drug release and targeted drug delivery can reduce systemic toxicity of chemotherapeutics by restricting drugs to the target organ and increasing the local concentration. As tumors and inflamed tissue are often surrounded by an acidic microenvironment, pH-responsive calcium carbonates (CaCO_3_) are promising vehicles for controlled drug delivery applications. The aim of this study was to evaluate the loading efficacy and release of a chemotherapeutic drug, Hydroxyurea (HU), into the crystal structure of calcite. Incorporation of HU did not alter the crystallinity, crystal size, or morphology of precipitated calcite crystals, as assessed by XRD and SEM. The amount of HU was quantified by High-Pressure Liquid Chromatography (HPLC) and showed that 6.7 ± 0.7 µg of HU could be for each milligram of calcite (0.016 mol% ± 0.002). In cell media, the optimal pH for controlled release was 5 (0.1 mg/mL released after 1 h). However, in vitro, pH below 6.5 was cytotoxic to human breast cancer cells (MCF-7). Direct contact studies, where particles were incubated with MCF-7 cells, showed that the amount of HU release from calcite was not high enough to kill the cell or arrest growth at pH 6.5. Pre-dissolved release studies, where the particles were pre-dissolved in acidic media to simulate complete drug release in vivo, pH neutralized, and exposed to the cells, showed that the amount of loaded HU reduced the survival/proliferation of MCF7. In conclusion, it is possible to integrate HU into the crystal structure of a calcite crystal and release the drug in vitro at concentrations that can slow the growth of cancer cells, without affecting calcite morphology and crystallinity. Further research is needed to investigate the in vivo behavior of the particles and whether the actual tumor pH is low enough to achieve complete drug release in vivo.

## 1. Introduction

Calcium carbonate (CaCO_3_ or Calcite) is an abundant and pervasive inorganic mineral used by many organisms to store ions and molecules [1]. Calcium carbonate has a large specific area and is biocompatible and pH-responsive [2,3,4]. These advantageous properties have made calcite the subject of extensive interest in pharmaceutical and biomaterials research. Calcite has been widely used as an excipient, treatment for heartburn, dietary supplement, and more by the pharmaceutical industry [5]. The pH-responsiveness of calcite allows it to act as a targeted, controlled release, drug delivery system [6]. When drug-loaded calcite is exposed to tumors or inflamed tissue, the acidic pH of the local microenvironment will cause calcite to dissolve and release drug, Ca^2+^ and CO_3_^2−^ ions. Of the three different polymorphs, calcite, aragonite, and vaterite, calcite is the most studied as it is more thermodynamically stable [7].

Precipitation methods are used to synthesize calcite as they are free of organic solvents and fairly simple. The controlled synthesis of calcite makes it possible to modify the size, morphology, shape, and functionalization. Previous studies have shown that it is possible to synthesize pH-responsive hybrid nano- and microparticles of various sizes and shapes, and also incorporate drugs via physicochemical adsorption [8,9]. Only one other study has reported that small molecules (amino acids) can be incorporated into individual calcite crystals [10]. As the amount of loaded drug increases, the morphology (i.e., rounded corners) and crystal structure are more likely to become distorted. Distortions in the crystal lattice can be measured with synchrotron X-ray diffraction, where the greatest lattice distortions are detected in the lattice plane (006). It is known that impurities can inhibit crystal growth by blocking active sites. Amino acids can adsorb to the surface of calcite, where calcium ions on the calcite surface associate with negatively charged carboxyl groups, and carbonate ions within calcite interact with the positively charged amino group [11].

In this study, we present a diffusion method for the entrapment of a small cytotoxic drug, Hydroxyurea (HU), into single crystals of calcite. An ideal synthesis method should have high loading efficiency so that crystals can store and release clinically effective concentrations of the drug, minimize the amount of calcite particles needed, and minimize off-target toxicity. The diffusion method allows the drug to be incorporated into a single crystal structure without affecting the overall crystal morphology. Crystal morphology is critical for determining the dissolution rate, surface energy, and potential for direct cell uptake (passive drug targeting) [12]. HU is a cytotoxic, anti-proliferative drug used to treat rapidly dividing diseases, such as sickle cell, leukemia, and breast cancers [13,14,15]. HU has a pKa of 10.15, meaning it will be neutrally charged below pH 10, and negatively charged above pH 10. At neutral pH, HU will not have any charged functional groups that can interact with the calcite surface, resulting in efficient entrapment of HU within the crystal structure via surface adsorption.

Previous studies investigated the possibility of synthesizing HU-loaded nanoparticles [16,17]. The nanoparticles were loaded with HU by attaching the drug to the particle’s surface, making it possible to adjust the dosage for different treatments. Azemati et al. studied human breast cancer cell (MCF-7 cells) proliferation after exposure to HU surface-loaded Fe_3_O_4_ nanoparticles [16]. Nano-sized particles have a larger surface area compared to microparticles, which makes it easier to load clinically effective doses. However, Azemati observed that attaching HU to the surface altered the particle morphology. Therefore, in this study, we investigated the possibility of incorporating HU into the crystal structure of micron-sized calcite crystals without altering crystal morphology. After synthesizing and characterizing HU-loaded calcite, their cytotoxicity/anti-proliferative effects were investigated using MCF-7 breast cancer cells.

## 2. Materials and Methods

### 2.1. Materials

Ammonium carbonate, ammonium acetate (HPLC degree), HU, and xanthyrol were purchased from Sigma Aldrich (Burlington, MA, USA). Calcium chloride was purchased from Fisher scientific and hydrochloric acid (HCl, 37% fuming) was purchased from Merck (Darmstadt, Germany).

### 2.2. Synthesis of Calcium Carbonate Powder

**Figure 1:**
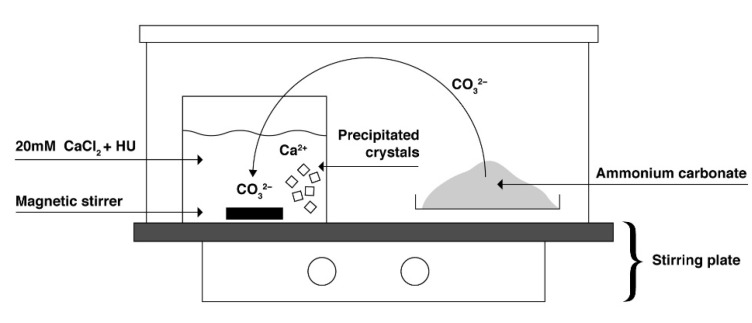
Illustration of the experimental setup for the synthesis of calcite.

As HU is classified as a cytotoxic drug, safety precautions were taken according to the Swedish work environment authority, article AFS 2005:5 [18]. The synthesis was performed similarly to the previously reported study by Kim et al. [10]. The synthesis was carried out in a closed box containing two liters of free volume (Figure 1). HU was dissolved in a 20 mM CaCl_2_ solution to obtain concentrations of either 400 mM or 800 mM. Further, 40 mL of the drug solution was poured into a Petri dish with a surface area of 56 cm^2^. A small magnetic stirrer was added and the Petri dish was sealed with perforated parafilm to allow precipitation to occur. Five grams of freshly crushed ammonium carbonate was added to another Petri dish. Both Petri dishes were placed into the box which was placed onto a magnetic stir plate. The synthesis was terminated after 48 h and the obtained precipitated crystals were filtrated and washed with water and ethanol. The powder was then air-dried for further analysis.

### 2.3. Drug Release Studies

After washing and drying, the powder was completely dissolved in 1 M HCl to determine the maximum release and loading efficiency. An aliquot was taken out and mixed with 700 µL of ethanol, 300 µL of 1 M HCl, and 50 µL of 0.02 M xanthyrol prior to the quantification with HPLC (RP-HPLC, Hitachi, Chiyoda, Japan).

Particle stability was determined by measuring drug release after incubating in phosphate buffer at pH 7.4. Additionally, 50 mg of particles were immersed in 10 mL of phosphate buffer and placed onto a shaker (IKAMAG^®^, IKA Werke GmbH & Co. KG, Staufen im Breisgau, Germany), at a speed of 50 rpm. An aliquot (300 µL) was taken out after 1 h, 1 day, 1 week, and 2 weeks.

### 2.4. HPLC

The quantification of HU was measured using reversed-phase high-performance liquid chromatography (RP-HPLC, Hitachi, Chiyoda, Japan) with UV detection (λ = 213 nm). Prior to injection in a column, the sample was mixed with 700 µL ethanol, 300 µL 1 M hydrochloric acid, and 50 µL 0.02 M xanthyrol in 1-propanol. HU is a small molecule that does not absorb UV and therefore, xanthyrol is added to act as a derivatization agent. An isocratic method was used with a mobile phase consisting of 50% 20 mM ammonium acetate (pH = 6.9) and 50% acetonitrile. The HPLC system was equipped with a Purospher^®^ STAR RP-C18 column (150 mm × 4.6 mm, 5 μm, Merck, Darmstadt, Germany).

### 2.5. Characterization

The morphology and size of the obtained crystals were studied with Scanning Electron microscopy (SEM; Zeiss Leo 1550 operated at 3 kV, Zeiss, Oberkochen, Germany). The samples were prepared by dispersing the powder in ethanol and evaporating onto carbon tape. Coating with Pt/Au was undertaken to avoid charging. The phase composition was determined by X-ray Diffraction (XRD; Bruker, D8 Advanced, Bruker, Billerica, MA, USA) by using CuKα (λ = 1.5418 Å). The diffractograms were recorded with a step size of 0.05, from 20–60° (2Θ) and a step time of 2 s.

### 2.6. Particles in Different pH

The dissolution of particles was investigated, visually and quantitatively, to determine what pH (1–6.5) was sufficient to dissolve each particle concentration (0.01–50 mg/mL) in order to achieve maximum drug release. Particle dissolution was conducted in well-plates and acidified DMEM/F12 cell media for up to 24 h in 37 °C. For visual investigation, images were taken with the microscope of the wells at different time points: 0, 1, and 24 h. To quantify the amount of particles dissolved, light absorbance was measured (λ = 560 nm) with the same dissolution time points.

### 2.7. Cell Culture

MCF-7 human breast cancer cells were purchased from ATCC. MCF-7 were cultured in DMEM/F12 (Gibco) with 10% fetal bovine serum (FBS) and 1% penicillin/streptomycin at 37 °C in a humidified atmosphere of 5% CO_2_. Complete media replacement was performed every 48 h. The cells were subcultured at 80% confluence and used within 6 passages from the thaw.

### 2.8. Treatment

MCF-7 cells were treated with different concentrations of calcite particles (direct contact). Pure calcite particles without drugs were selected as the control group. Particles were sterilized by washing in 70% ethanol, followed by resuspension in fresh cell media. Cells were treated with different concentrations of particles and the proliferation was investigated after 1 and 3 days.

#### Treatment with Particle Solution

The calcite particles were dissolved in 1 M hydrochloric acid, neutralized with 1 M sodium hydroxide, and diluted with DMEM/F12 media (1:10). The obtained particle solution was used to treat the cells and the proliferation was investigated after one and three days, respectively.

### 2.9. Survival/Proliferation

Cell survival/proliferation was determined with Alamar blue. Cells were seeded at 1, 2, or 4 × 10^4^ cells/well in 96-well tissue culture-treated plates. After 24 h, the media was replaced with media containing calcite particles, loaded with or without HU over a concentration range of 100 µg/mL to 10 mg/mL. After incubating cells with particles for 24 or 48 h, the media was replaced with 150 µL of 10% Alamar blue solution in fresh media. The plates were incubated at 37 °C for one hour before transferring 100 µL of the supernatant to a black 96-well plate. The fluorescence was detected at 570 nm excitation and 590 nm emission on a microplate reader (Infinite M200, Tekan, Switzerland).

### 2.10. Statistical Analysis

Data are reported as mean ± standard deviation. For Figure 9, IBM SPSS Statistics (IBM Corp. New York, NY, USA) was used to perform a one-way analysis of variance (ANOVA) with Tukey post hoc tests (assumptions of normality and homogeneity of variance were not violated) to identify the difference between groups. The level of significance was set to *p* = 0.05.

## 3. Results

### 3.1. Characterization

**Figure 2:**
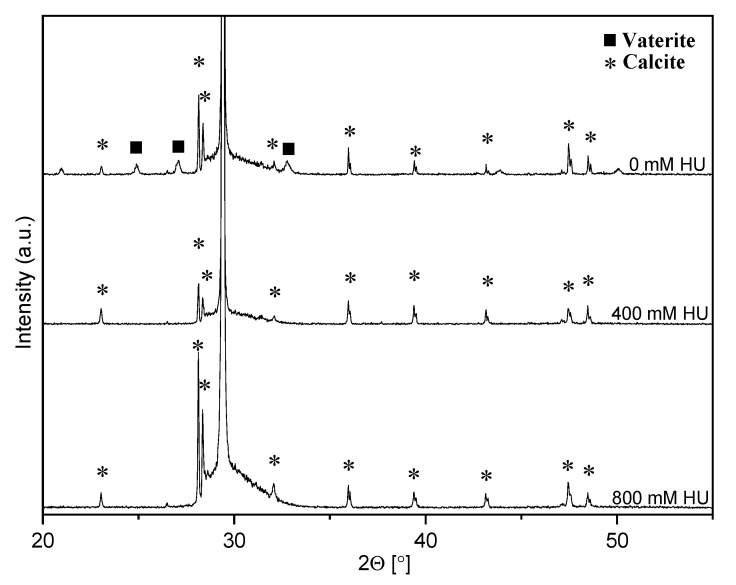
Diffraction patterns for calcite with and without drug. Both vaterite (▪) and calcite (*) were formed with no drug present while only calcite was formed in the presence of hydroxyurea.

**Figure 3:**
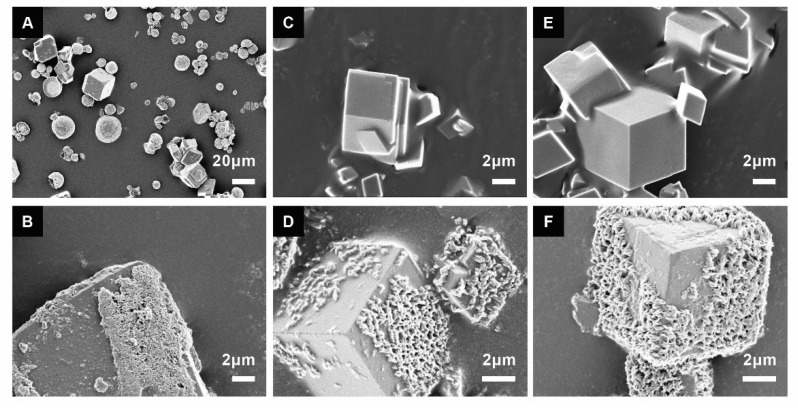
Calcite crystals with different concentrations of the drug before (**A**,**C**,**E**) and after two weeks in PBS solution (**B**,**D**,**F**); 0 mM hydroxyurea (**A**,**B**), 400 mM hydroxyurea (**C**,**D**), and 800 mM hydroxyurea (**E**,**F**). Hydroxyapatite was formed on the surface after two weeks in PBS for all formulations. In the non-drug-loaded calcite synthesis group, two phases were observed: the cubic-shaped calcite and the spherical-shaped vaterite (**A**).

XRD spectra and SEM showed that vaterite and calcite both formed when no drug was present (Figure 2 and Figure 3). The addition of HU restricted the formation of crystalline material only to calcite crystals (Figure 2 and Figure 3). Due to peak broadening, the degree of crystallinity showed only a slight difference between the particles with and without HU: 87% for particles without HU and 83% for the particles with HU. The crystals that were used for studying prolonged release (2 weeks) in PBS solution showed the formation of hydroxyapatite on the surface, Figure 3.

### 3.2. Drug Loading Efficiency

The pH of the synthesis solution was measured before and after the synthesis reaction, Table 1. The pH decreased with increasing amounts of HU while the pH after the termination of synthesis was the same for all concentrations.

**Figure 4:**
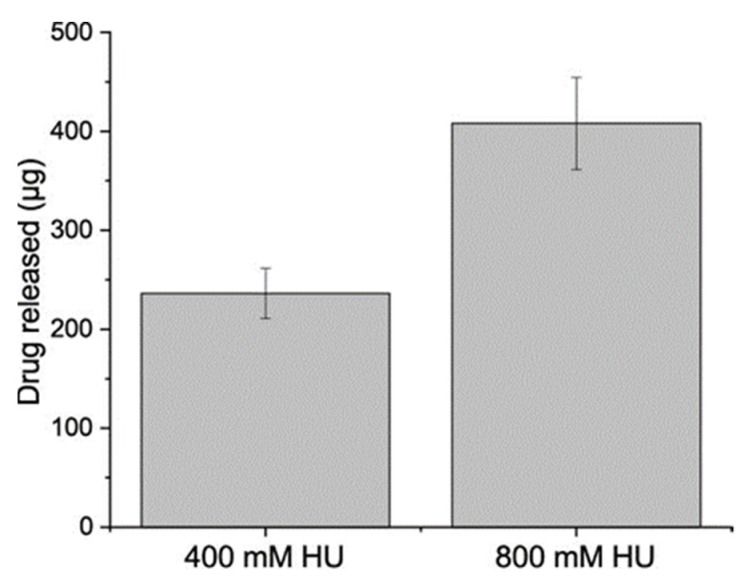
Amount of HU recovered after washing with water and ethanol. The particles were dissolved in 1 M HCl, (Mean ± SD, *n* = 3).

Higher initial concentrations of HU resulted in a higher amount of loaded HU, Figure 4. The amount of drug detected are shown in Table 2, where the loading efficiency (mol%) was calculated from the release concentration dived by the starting concentration. Drug release at pH 7.4 (PBS solution) was conducted for two weeks. For all formulations, no drug was detected at pH 7.4, confirming that drug release did not occur at neutral pH.

### 3.3. Drug Release Conditions

**Figure 5:**
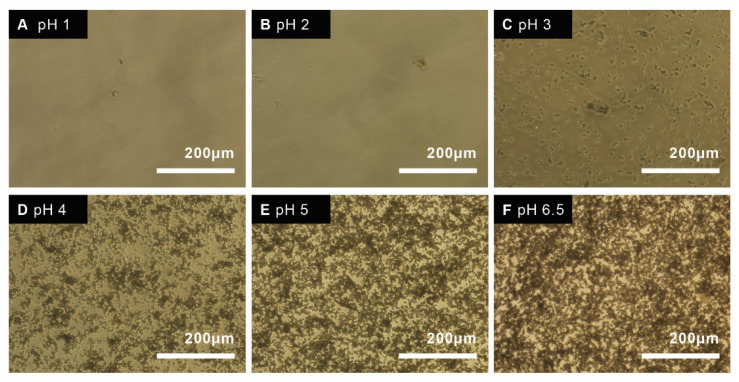
Microscope image of 1 mg/mL particles in acidified DMEM cell media, incubated in 37 °C at different pH; pH 1 (**A**), pH 2 (**B**), pH 3 (**C**), pH 4 (**D**), pH 5 (**E**), and pH 6.5 (**F**) at T = 0 h.

The optimal pH for dissolving the calcite particle is below pH of 4, which resulted in almost instantaneous dissolution (Figure 5).

**Figure 6:**
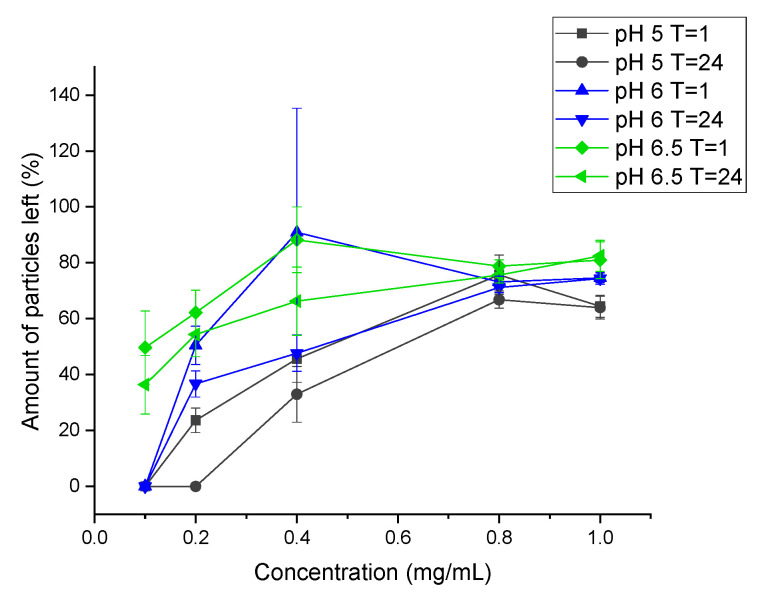
Dissolution of particles measured by absorbance at different time points and pH. (Mean ± SD, *n* = 3).

However, the pH must remain close to physiological pH (7.4) for cell studies, therefore the rate of dissolution was determined for different pH close to 7.4 that allowed for release and cell survival (Figure 6). The amount of dissolved particles was quantified by measuring the absorbance of particles at different time points and different pH. Particles were fully dissolved after 1 h at 0.1 mg/mL at pH 5 and 6.

### 3.4. Cell Viability in Direct Contact during Drug Release

**Figure 7:**
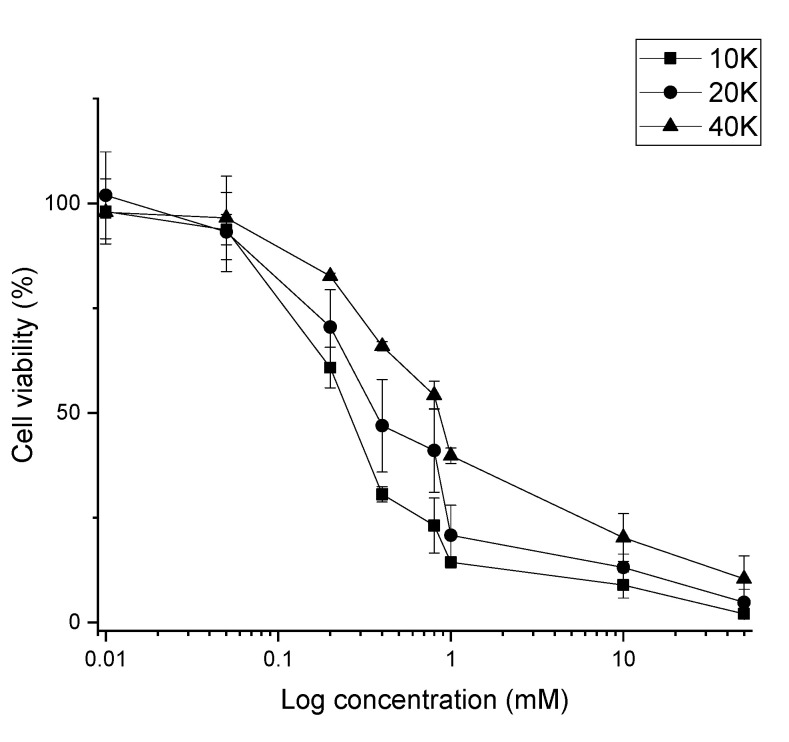
Cell viability assay for determination of EC50 of Hydroxyurea at three different cell concentrations (1 × 10^4^, 2 × 10^4^, and 4 × 10^4^ cells/well). (Mean ± SD, *n* = 3).

Cell viability studies are typically conducted on cells that are actively dividing in the linear range of logarithmic division. However, tumors are typically dense, highly populous three-dimensional cultures. The EC50 value for a given drug can differ greatly between cell concentrations as lower density cultures are more susceptible to drug-induced toxicity. Therefore, HU toxicity was validated using three different cell concentrations, 1 × 10^4^, 2 × 10^4^, and 4 × 10^4^ cells/well (Figure 7). The observed EC50 values of HU in MCF-7 cells was 0.2 mM for cells cultured at 1 × 10^4^/well, 0.4 mM for cells cultured at 2 × 10^4^/well, and 0.8 mM cells cultured at 4 × 10^4^/well.

**Figure 8:**
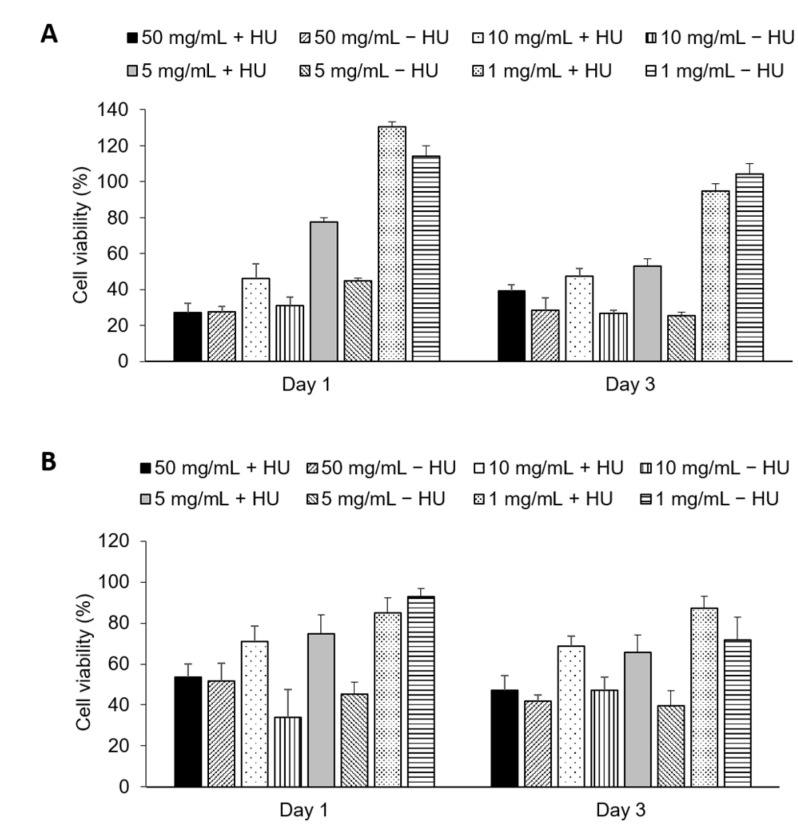
Cell viability after treating 20,000 cells/well (**A**) and 40,000 cells/well (**B**). The cells were treated with 50 mg/mL, 5 mg/mL, 10 mg/mL, and 1 mg/mL. For each concentration, particles with (+) and without (−) Hydroxyurea was tested. (Mean ± SD, *n* = 6).

Cell viability was determined with Alamar blue assay. Calcite drug-loaded particles reduced viability by up to 50–70% (50 mg/mL, Figure 8), though this appears to be due to direct toxicity caused by the particles, as non-drug loaded particles had comparable rates of survival. Interestingly, empty calcite particles were more toxic than drug-loaded calcite at sub-confluent cell densities (2–4 × 10^4^ cells/well, 1–10 mg/mL, Figure 8).

#### Cell Viability with Pre-Dissolved Drug Release

**Figure 9:**
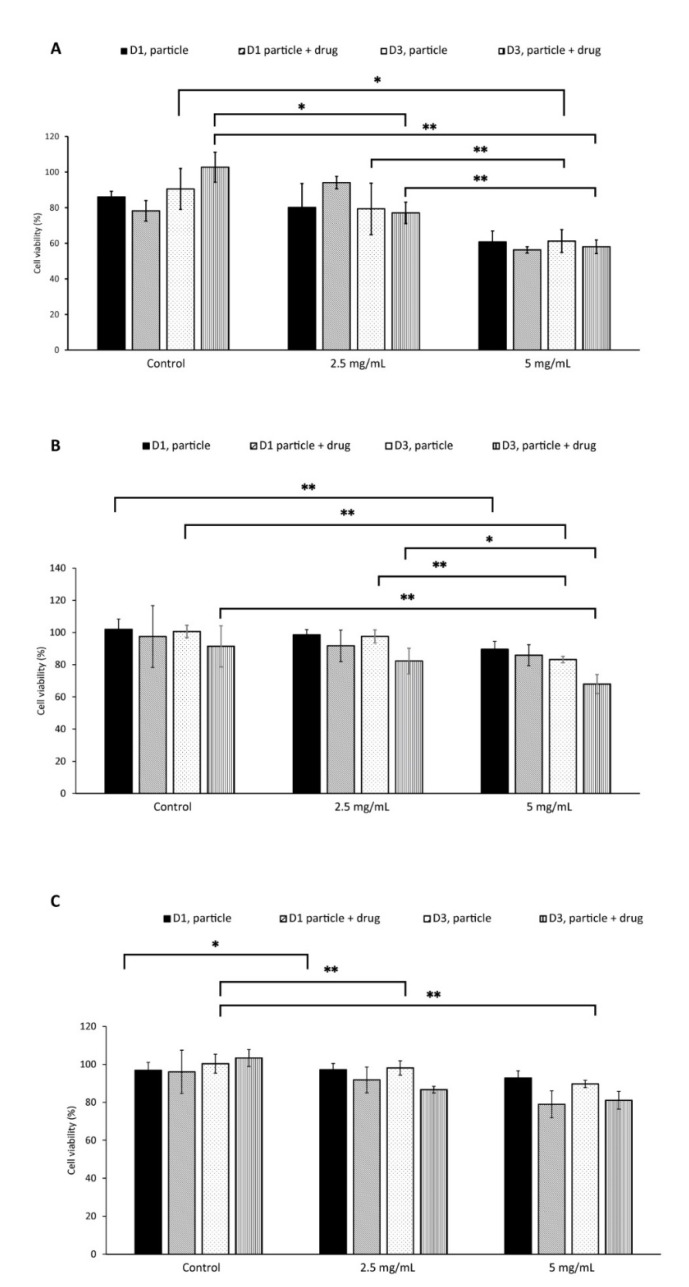
Cell viability after particle solution treatment for 10,000 cell/well (**A**), 20,000 cells/well (**B**), and 40,000 cells/well (**C**). The cells were treated with 2.5 mg/mL and 5 mg/mL. For each concentration particle with (+) and without (−) Hydroxyurea was tested. All *p* values listed used the parametric ANOVA and Tukey posthoc analysis, *n* = 6, * indicates *p* < 0.05, and ** indicates *p* < 0.01.

The particles were pre-dissolved in 1 M hydrochloric acid, neutralized with 1 M sodium hydroxide, and diluted with DMEM/F12 media (1:10). Cells were treated with dissolved particles, with and without drug at two concentrations 2.5 mg/mL and 5 mg/mL. The dissolved particles containing HU had lower cell viability compared to the particles without HU, meaning that HU does have an effect on the cells and that the limiting step is the slow dissolution of particles (Figure 9).

## 4. Discussion

In this study, we demonstrated for the first time that calcite single crystals containing HU can be synthesized. Different experimental approaches were used to confirm the incorporation of the drug, including immediate release when dissolved at acidic pH levels and slow release at physiological pH for two weeks.

Previous attempts have been made to load different drugs and molecules into calcite crystals. The majority of these studies resulted in hybrid microparticles, where the drug molecule is adsorbed onto the surface [8,9]. A study by Kim et al. showed entrapment of 6.9 mol% of amino acids into the crystal structure of calcite [10]. However, the addition of amino acids resulted in a change of morphology where the calcite corners were smoothed out. It is known that amino acids tend to adsorb to the calcite surface as the amino group can interact with the carbonate groups and the carboxylic group can interact with the calcium ions [11]. In the present study, HU is used, which is a slightly basic molecule with a pKa of 10.15. The functional groups and the basicity of HU imply that no adsorption would occur at the calcite crystal surface, meaning that all of the HU will be within the crystal structure. The total amount of loaded HU for 400 mM and 800 mM was calculated to be 0.019 mol% and 0.016 mol%, respectively. These amounts are significantly lower compared to Kim et al., however in this study, the drug is incorporated into the crystal structure without any adsorption to the surface.

Any potential non-specific drug adsorbed to the particle surface was thoroughly removed using water and ethanol washes prior to all tests, as HU is highly soluble in water. The amount of loaded HU was analyzed by dissolving the calcite in HCl, which showed that increasing the starting concentration of HU in the synthesis solution leads to a higher amount of loaded HU (Figure 4). However, the lower initial concentration of HU during precipitation (400 mM) was shown to be more efficient, i.e., a higher mol% (0.019 mol%) of entrapped HU, compared to the higher concentration (800 mM, 0.016 mol%), Table 2. This can be explained by the saturation of the drug in the solution, i.e., the higher starting concentration is not as efficient as the lower concentration. Drug release was evaluated over two weeks, in PBS (pH 7.4) solution, which resulted in no drug release at physiological pH. These results confirmed that our calcite HU-loaded particles are pH-responsive.

The obtained XRD data showed a peak broadening for all samples and the calculated degree of crystallinity showed slightly lower crystallinity in the particles with HU compared (83%) to the particles without the HU (87%). Peak broadening might be caused by various reasons such as crystal size, micro-strains, etc. Kim et al. analyzed peak broadening after loading of amino acids and found that drug incorporation leads to peak broadening due to strains in the structure [10]. In our case, the crystals are micronized, and no amorphous particles could be seen in the SEM, which rules out crystal size as being the cause of peak broadening. It is possible that the peak broadening is due to micro-strains that are caused by the incorporation of HU. The morphology of the particles after prolonged exposure to PBS for two weeks was investigated by SEM, which showed the formation of hydroxyapatite on calcite crystals (Figure 3). Previous studies showed that calcium ions will slowly dissolute from calcite_,_ making it possible for the phosphate ions to reprecipitate hydroxyapatite on the slightly basic surface of calcite [19]. During synthesis, HU decreased the pH of drug-loaded calcite solutions (Table 1). Paradoxically, while vaterite forms at room temperature and in lower pH [20,21], vaterite did not form in the lower pH HU-loaded calcite solutions. This supports the notion that HU may stabilize calcite against dissolution and pH-related effects.

The synthesized particles, both with and without drugs, were tested on human breast cancer cells (MCF-7). The EC50 value for HU was reported to be approximately 200 µM for breast cancer cell lines [22,23]. The EC50 value was confirmed in this study, 200 µM of HU for 1 × 10^4^ cells/well, 400 µM for 2 × 10^4^ cells/well, and 800 µM for 4 × 10^4^ cells/well. Due to HU’s high EC50, a higher concentration of HU is needed to reduce cell proliferation. Theoretically, 10 mg of particles must be dissolved (100% release) per mL of fluid to reach the EC50 for a cell concentration of 40 K. The solubility of the particles was investigated in different pH close to physiological pH, which suggested that a maximum of 0.1 mg/mL of particles could be dissolved within 1 h (Figure 6). As the pH needed to completely dissolve this amount of drug-loaded calcite (pH 2–3) is far below physiological pH, the empirical solubility of the particles was investigated in different pH media closer to physiological pH (pH 5–7) and we found that, in vitro, in situ dissolution was feasible only for concentrations of calcite <1 mg/mL (Figure 5 and Figure 6). Drug-loaded particles were less toxic than non-loaded calcite (Figure 8). One explanation could be that drug-loaded HU particles are not dissolving quickly enough to release sufficient drugs. Therefore, an additional in vitro test was performed where the synthesized particles, with and without drugs, were dissolved, neutralized, and diluted with cell media. The obtained particle solutions, 2.5 mg/mL and 5 mg/mL were tested on cells (Figure 9). The result indicated that HU does affect cell proliferation, meaning that the release of loaded HU is mostly limited by the dissolution rate of the crystal at neutral pH (Figure 5 and Figure 6). The lowest cell concentration (1 × 10^4^ cells/well) has an EC50 of 200 µM HU which translates into 2.5 mg/mL of particle solution. The effect of HU could not be observed at this concentration, i.e., the same proliferation could be observed both with and without drugs. It is possible that the presence of high calcium ion concentration caused the cells to die [24]. The highest effect of HU was observed in the concentration of 20,000 cells/well where cell death of 70% was achieved after three days of treatment with 5 mg/mL of particle solution.

One major limitation of the present study is that various mechanisms underlying drug delivery could not be investigated. The dissolution mechanism and kinetic rate were not investigated, which means the authors cannot conclusively explain why the non-drug loaded particles were more toxic at higher concentrations than drug-loaded particles. Unfortunately, in vitro cultures cannot effectively mimic the in vivo tumor microenvironment (pH 5, hypoxic, very high cell density, etc.), therefore it is necessary to assess whether HU-loaded particles could be an effective delivery vehicle using in vivo models.

Previous studies have investigated the in vitro properties of HU when attached to the surface of nano-sized particles [16,17]. While attaching HU to the surface makes it easier to add the desired amount of drug needed for treatment, this often completely changes the morphology of the particles. Therefore, the results obtained in this study are of significant importance, as we have shown that it is possible to add clinically relevant amounts of drug into the calcite crystal structure without negatively affecting the morphology and crystal size. This result is significant, as it has not been reported for calcite microparticles and molecules larger than amino acids.

To summarize, this study shows that it is possible to synthesize calcite, micron-sized, single crystals containing HU within the crystal structure, without changing their morphology. These findings open the possibilities to investigate multiple drug molecules that possess higher pKa with functional groups that could or could not interact with the calcite surface. These results make it possible to synthesize inorganic material that can function as both bulk material and pharmaceutical. The main obstacle is the low EC50 value of HU, meaning that the amount of loaded and released HU is not sufficient enough to kill the cells in vitro, when the media pH is strictly buffered at 6.5–7.4, but may be effective in vivo where the local low pH could increase the dissolution of particles. Most importantly, we successfully loaded HU in sufficient quantities to achieve growth arrest with a clinically relevant dose (5 mg/mL). Further investigations need to be undertaken to find a more suitable drug that has a lower EC50 value. Furthermore, the in vitro properties were investigated with MCF-7 cells. The observed cell death was similar between crystals with and without HU. A possible explanation for this might be the high concentration of calcium released when calcite is dissolving in the slightly acidic cell media.

## 5. Conclusions

In this study, an ammonium diffusion method was used to synthesize calcite single crystals with HU entrapped within the crystal structure. Both SEM and XRD confirmed the formation of crystalline calcite for both formulations. HU-loaded calcite displayed controlled release properties, with drug release occurring only below pH 5. Furthermore, the crystal morphology was investigated before and after release showing the formation of hydroxyapatite when the crystals were exposed to PBS. The calcite crystal’s effect on human breast cancer cell viability was also assessed. The high EC50 and the amount of HU released from the particles were not sufficient to kill the cancer cells when in direct contact due to slow dissolution in pH-neutral cell media. However, HU released from calcium carbonate particles does decrease cell proliferation when the particle dissolution rate is sufficiently high. These findings are of great importance for future studies involving incorporating drugs into single crystalline material such as calcite. Further investigations need to be performed to optimize the crystals’ dissolution rate.

## Figures and Tables

**Table 1:** pH measurements of starting concentration for the synthesis.

Concentration of Hydroxyurea	Initial pH	Final pH
0 mM	6.99	9.20
400 mM	6.58	9.03
800 mM	6.32	9.87

**Table 2:** Summary of collected data and calculations of loading properties for the two synthesis conditions (400 mM HU and 800 mM HU).

Synthesis Regimen	Amount of HU (µg)	Amount HU/Calcite (µg/mg)	Loading Efficiency (mol%)
400 mM	236.1 ± 25.5	3.6 ± 0.3	0.019 ± 0.002
800 mM	408.0 ± 46.5	6.7 ± 0.7	0.016 ± 0.002

## Data Availability

Not applicable.
